# Novel ERα positive breast cancer model with estrogen independent growth in the bone microenvironment

**DOI:** 10.18632/oncotarget.10443

**Published:** 2016-07-06

**Authors:** Aude-Hélène Capietto, Szeman Ruby Chan, Biancamaria Ricci, Julie A. Allen, Xinming Su, Deborah V Novack, Robert D. Schreiber, Roberta Faccio

**Affiliations:** ^1^ Department of Orthopedics, Washington University School of Medicine, St. Louis, MO, USA; ^2^ Department of Pathology and Immunology, Washington University School of Medicine, St. Louis, MO, USA; ^3^ Department of Medicine, Washington University School of Medicine, St. Louis, MO, USA; ^4^ Present address: Genentech, South San Francisco, CA, USA; ^5^ Present address: Janssen Research and Development, Johnson and Johnson, Spring House, PA, USA

**Keywords:** skeletal metastasis, breast cancer, endocrine resistance, bone, hormone resistance

## Abstract

Despite successful therapeutic options for estrogen receptor-α (ERα)+ breast cancer, resistance to endocrine therapy frequently occurs leading to tumor recurrence. In addition to intrinsic changes in the cancer cells, herein we demonstrate that tumor cell-microenvironment interactions can drive recurrence at specific sites. By using two ERα+ cell lines derived from spontaneous mammary carcinomas in STAT1−/− mice (SSM2, SSM3), we establish that the bone microenvironment offers growth advantage over primary site or lung in the absence of ovarian hormones. While SSM3 did not engraft at primary and skeletal locations in the absence of estrogen, SSM2 selectively grew in bone of ovariectomized mice and following administration of aromatase inhibitors. However, SSM2 growth remained hormone-dependent at extraskeletal sites. Unexpectedly, bone-residing SSM2 cells retained ERα expression and JAK2/STAT3 activation regardless of the hormonal status. These data position the bone microenvironment as a unique site for acquisition of tumor/estrogen independency and identify the first ERα+ hormone-independent tumor model in immunocompetent mice.

## INTRODUCTION

The skeleton is the most common site of breast cancer metastasis [1]. Breast cancer is the most common malignancy and the leading cause of cancer death among women worldwide [2]. Nearly two thirds of breast tumors overexpress the receptors for estrogen (ERα) and/or progesterone (PR), and ERα+/PR+ tumors have a greater propensity to metastasize to bone than to the viscera [3, 4]. Although the prognosis for patients with skeletal metastases is better than those with extra-skeletal metastases [5] median survival is only ~2 years [5]. In addition, bone metastases are associated with significant morbidity due to the development of skeletal related events (SREs), defined as pathological fractures, spinal cord compression, hypercalcemia and bone pain [5] secondary to osteoclast-mediated bone resorption [6]. The standard of care for ERα+ breast tumors is either to inhibit ER signaling using selective ER modulators or to deprive the tumors of estradiol by ovarian ablation (OVX) or aromatase inhibition. Furthermore, patients with skeletal manifestations are also treated with anti-resorptive agents. While combination therapies with anti-resorptive agents, such as zoledronic acid, successfully decrease SREs [1], recurrence is still high [7, 8] often due to acquired resistance to anti-hormone therapies.

Loss of ER expression is highly predictive of acquired resistance to anti-hormone therapy associated with progressive disease [9]. However, patients who relapse on aromatase inhibitors can also retain functional ER expression [10]. Therefore, down-regulation of ER alone cannot explain the estrogen-independent growth of ER+ tumors. Other mechanisms, such as ligand-independent growth factor receptor signaling pathways, have been shown to lead to endocrine resistance [11, 12]. Recent reports suggest that interactions between tumor cells and cells in the local microenvironment, such as perivascular macrophages, are important for intravasation thus increasing the risk of metastases at distant sites in ER+ tumors [13]. However, the mechanisms of ER+ breast cancer relapse in bone or other sites in spite of initial response to anti-hormone therapy remain to be elucidated.

Currently we lack ER+ experimental metastatic models that become resistant to estrogen-deprivation. Several human ER+ breast cancer cell lines, such as MCF-7, T47D and ZR75 cells, can develop estrogen-independent growth after long-term drug exposure *in vitro* and thus be used as breast cancer estrogen-independent tumor models *in vivo* [11, 12]. However, these cell lines are poorly invasive and rarely metastasize. Resistance to estrogen-deprivation is not acquired during tumor progression *in vivo*. Furthermore, because of their human origin, these tumors only grow in immunodeficient mice, limiting our understanding of the contribution of immune cells to tumor relapse.

In this study we have identified two new ERα+/PR+ mouse mammary tumor cell lines, which develop skeletal metastases in immunocompetent animals. These tumor cell lines, called SSM2 and SSM3, have been derived from spontaneous luminal mammary carcinomas in STAT1−/− mice [14]. Considering that STAT1 expression has been associated with improved outcome prediction in patients with breast cancer [15] and STAT1 levels were found either undetectable or very low in 45% of ERα+ breast cancer patients [14], these tumor cell lines represent a clinically relevant ERα+/PR+ mammary tumor model. Furthermore, SSM2 and SSM3 cells maintain a similar luminal phenotype to human ER+ luminal breast cancer [14] and retain estrogen-dependent growth when re-inoculated in the mammary fat pad of immunocompetent WT mice [14]. We now find that while SSM3 cells do not grow in bone of OVX animals, SSM2 cells become resistant to hormone-deprivation in the bone microenvironment. To our knowledge, this is the first preclinical model describing hormone-independent growth of ERα+/PR+ breast tumor cells in the context of bone metastasis in immunocompetent animals.

## RESULTS

### ERα+/PR+ SSM2 and ERα+/PR+ SSM3 breast tumor cells induce osteolytic lesions in immunocompetent animals

**Figure 1:**
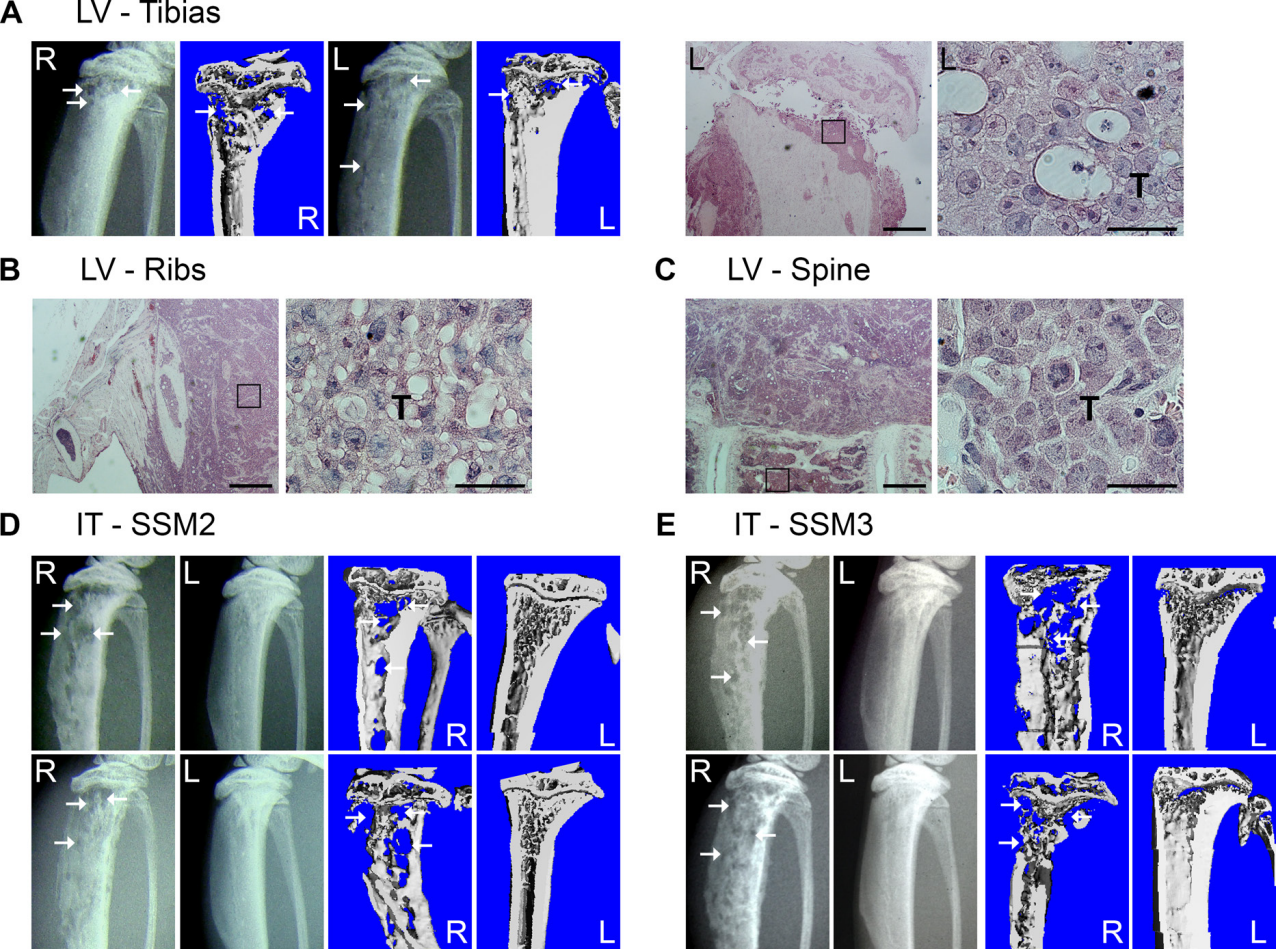
SSM2 and SSM3 cells develop osteolytic metastasis in immunocompetent female mice (**A–C**) 10^5^ SSM2 cells were injected into the left ventricle (LV) of WT immunocompetent female mice (*n* = 5) and development of bone metastasis was monitored by X-ray and viva-CT scans in the tibias, or by histology of the tibias, ribs and spine at time of sacrifice. (A) Representative X-ray images and viva-CT scans of the right (R) and left (L) tibias 48 days post-tumor cell injection. Arrows pointing to osteolytic lesions. H&E staining (original magnification, 2×. Scale bar = 500 μm. Insets showing SSM2 tumor cells at 60 × magnification) of the left tibia (A), ribs (B) and spine (C). Presence of SSM2 cells is indicated by T. (**D–E**) 10^5^ SSM2 or SSM3 cells were injected into the right tibia (IT) of WT female mice (*n* = 6/group). Representative X-ray and viva-CT images of the tumor-injected tibia (R) and the control non-injected tibia (L) 28 days post tumor inoculation. Arrows pointing to osteolytic lesions.

SSM2 and SSM3 breast cancer cell lines have been derived from primary tumors in STAT1−/− mice and express ERα and PR [14]. Because patients with ERα+/PR+ breast tumors often develop bone metastases, we first sought to determine whether ERα+/PR+ SSM cells have the capacity to colonize and grow in the bone microenvironment of naïve female mice. 10^5^ SSM2 cells were injected into the left ventricle of 129S6/SvEv (WT) immunocompetent female mice, and development of osteolytic lesions was monitored every other week by radiography and confirmed by viva-CT analysis before sacrifice. Areas of bone erosion were observed in tibias by 48 days post tumor cell injection and the presence of tumor cells confirmed by histology (Figure 1A). Metastatic dissemination to spine and ribs was also detected (Figure 1B, 1C), demonstrating the great propensity of these tumor cells to colonize the skeleton. Because the mice were often paralyzed, we turned to the intratibial injection to specifically study the growth of SSM2 and SSM3 cells in bone. 10^5^ SSM2 or SSM3 cells were injected directly into the right tibias (R) of WT female mice and development of osteolytic lesions was monitored every other week by X-rays and confirmed by viva-CT analysis before sacrifice. The non-injected left tibias (L) were used as control (Figure 1D, 1E). Both SSM2 and SSM3 cells grew in the bone and induced osteolytic lesions within 28 days post tumor injection in 100% of injected female animals (Figure 1D, 1E). No lytic lesions were observed in the non-injected tibias. To our knowledge, SSM2 and SSM3 represent the first ERα+/PR+ murine breast cancer model of bone metastasis in immunocompetent mice.

### ERα+/PR+ SSM2 cells grow in bone independent of ovarian hormones

**Figure 2:**
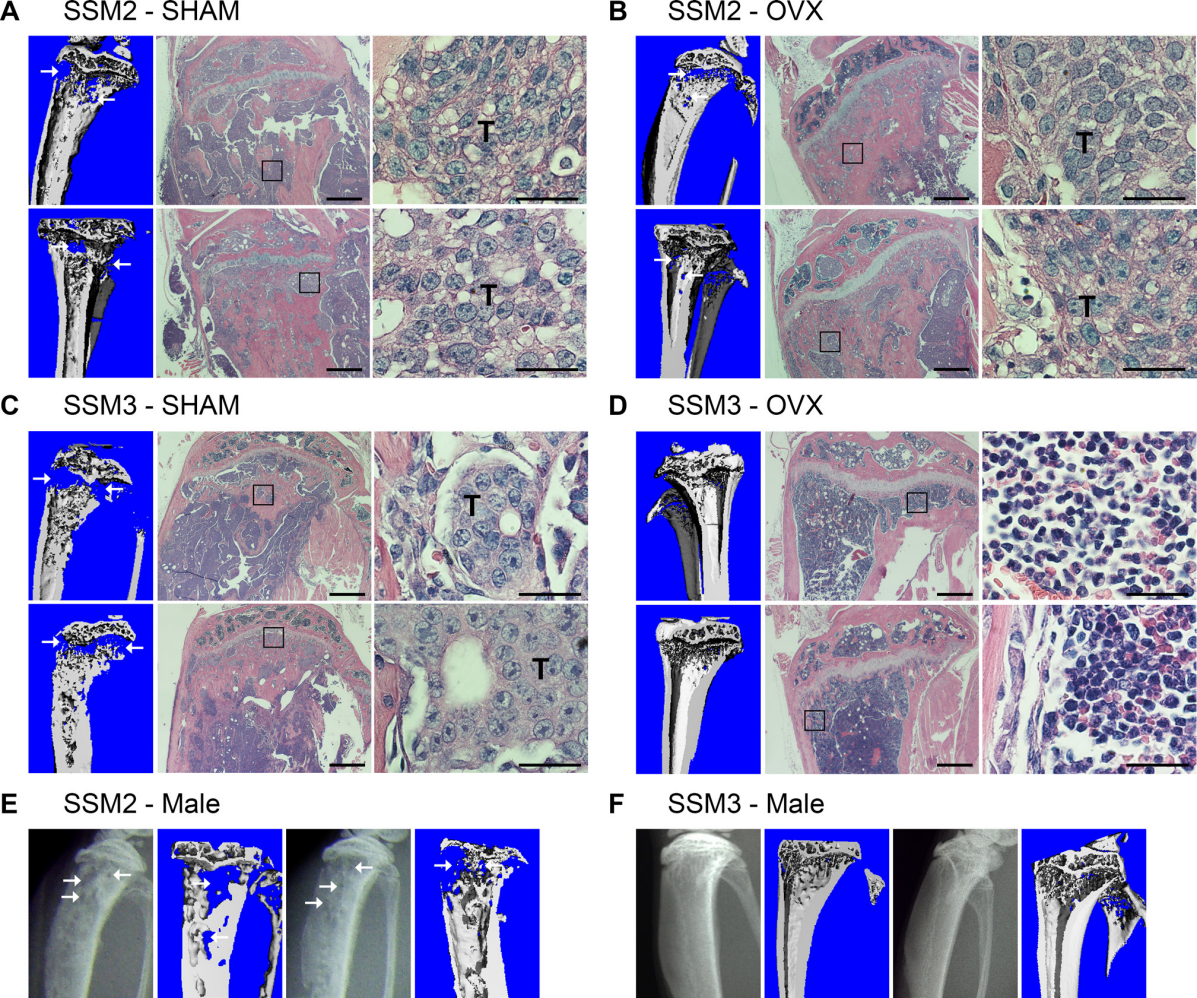
ERα+/PR+ SSM2 but not SSM3 cells grow in bone independent of ovarian hormones (**A–D**) 10^5^ SSM2 or SSM3 cells were injected into the right tibia of WT female mice (*n* = 6/group), one week after SHAM (A, C) or OVX surgery (B, D). 2 out of 6 representative images of viva-CT and H&E staining sections 28 days post tumor cell inoculation (original magnification, 2x. Scale bar = 500 μm. Insets showing the presence of tumor cells at 60x magnification, scale bar = 25 μm). (D) SSM3-bearing OVX mice were monitored for the absence of tumor-induced osteolysis until 56 days post tumor cell inoculation. Arrows pointing to osteolytic lesions. Presence of SSM2 or SSM3 cells is indicated by T. (**E**, **F**) 10^5^ SSM2 or SSM3 cells were injected into the right tibia of WT male mice (*n* = 5/group). 2 out of 5 X-ray images and viva-CT scans are shown. Osteolytic lesions are depicted by arrows. (E) SSM2-injected mice were sacrificed starting 28 days post tumor cell inoculation. (F) SSM3-injected mice were monitored for the absence of tumor-induced osteolysis until 85 days post-tumor inoculation.

To determine whether the bone microenvironment has any effects on hormone-dependency of tumor growth, ERα+/PR+ SSM2 and SSM3 tumor cell lines were injected into the tibias of WT mice one week after OVX or SHAM surgery as control. Surprisingly, SSM2 cells grew in bone independently of estrogen in 6/6 animals, as shown by presence of tumor cells by histology, and developed osteolytic lesions in both OVX and SHAM-operated mice, as observed by viva-CT scans (Figure 2A, 2B). By contrast, ERα+/PR+ SSM3 did not grow in the bones of OVX mice, while developing bone lesions in SHAM-operated animals (Figure 2C, 2D). Thus, SSM2 acquire the ability to grow in bone independent of ovarian hormones, whereas SSM3 maintain estrogen-dependent growth in bone similarly to mammary fat pads.

Because ovariectomy promotes osteoclast activation due to a decreased in estrogen levels, we further evaluated the estrogen-independent growth capacity of SSM2 cells after their inoculation into the tibias of male mice. Similar to the OVX model, development of SSM2 osteolytic lesions was observed by X-rays starting 28 days post tumor cell injection in 5 out of 5 male mice, and was confirmed by viva-CT analysis before sacrifice (Figure 2E). In contrast to SSM2, SSM3 cells did not show signs of lytic lesions by X-rays or viva-CT analysis up to 85 days post tumor inoculation in male mice (Figure 2F).

**Figure 3:**
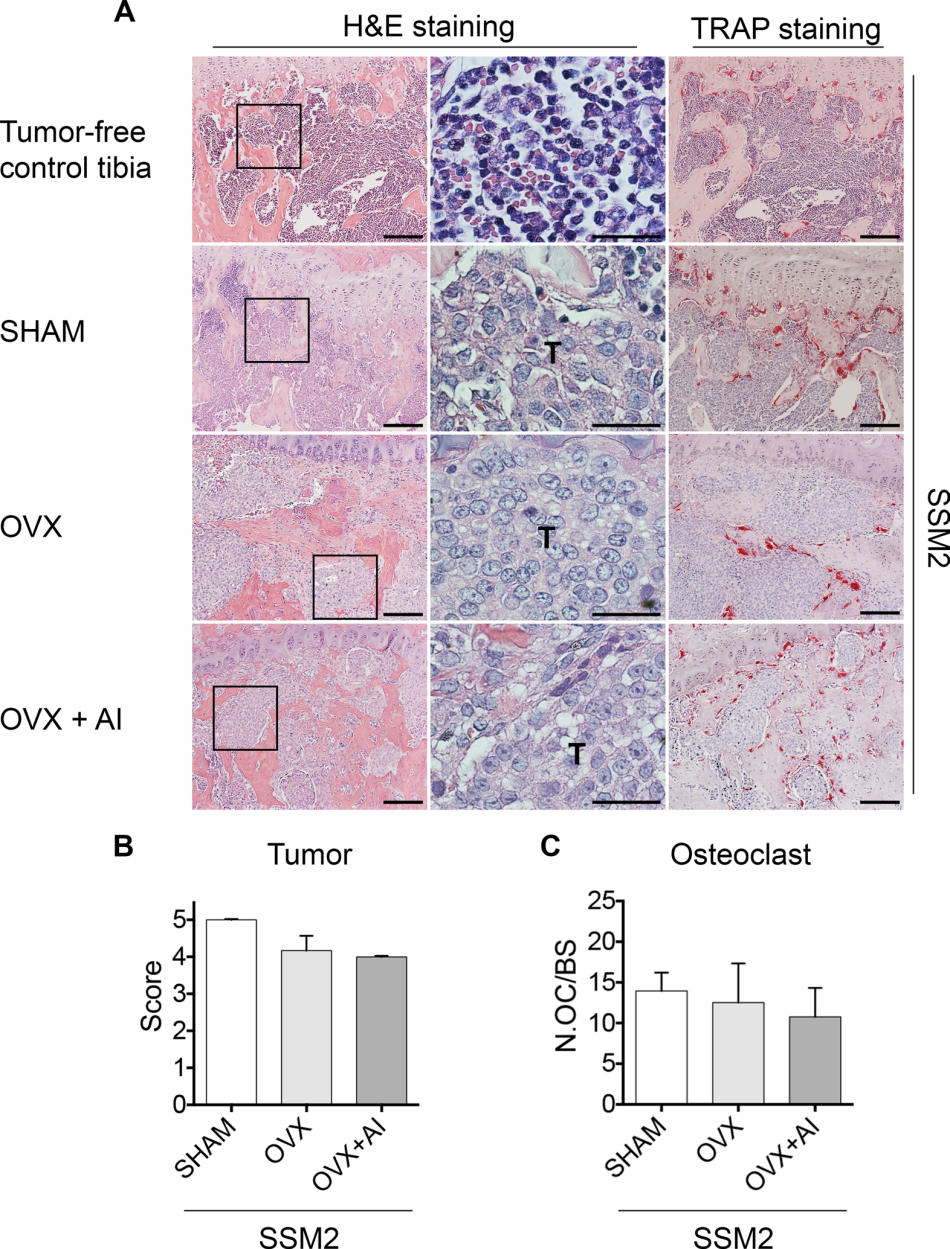
inhibition of peripheral estrogen synthesis does not prevent development of SSM2 tumor in bone (**A**) Representative H&E staining sections (original magnification, 10×. Scale bar = 100 μm. 60× magnification, scale bar = 25 μm) at 4 weeks post tumor cell inoculation. Presence of tumor cells is indicated by T. 10^5^ SSM2 cells were injected into the right tibia of WT female mice (*n* = 3/ group), one week after SHAM- or OVX- surgery. In addition to OVX, one group (*n* = 3/group) received aromatase inhibitor (AI) for 4 weeks. Representative TRAP staining is shown (original magnification, 10×. Scale bar = 100 μm). As control, representative H&E and TRAP staining is shown on sections from tumor-free tibia. (**B**) Tumor grade score from 0 to 5 showing displacement of marrow by tumor, with 0 indicating no tumor and 5 100% tumor in the bone marrow. Data are represented as mean +/− sem. No statistical differences were observed between groups. (**C**) Number of osteoclasts (OC) per bone surface is shown for SHAM, OVX and OVX + AI groups. Data are represented as mean +/− sem. No statistical differences were observed between groups.

To further determine whether increased sensitivity to limited amounts of estrogen could allow SSM2 tumor engraftment in male mice and/or following OVX, we administered the aromatase inhibitor Letrozole (AI, 10 ug/ day) to ovariectomized animals for 4 weeks and examined the extent of tumor growth in bone by histological analysis. As shown in Figure 3A, tumor cells occupied the majority of the bone marrow space with only few marrow cells visible by histology and because of the severe tumor-induced cortical bone erosion, quantification of tumor area versus total bone area was not possible. Therefore, tumor injected tibias were graded from 0 to 5 where 0 indicates no tumor and 5 indicates bone marrow entirely replaced by tumor cells. We found comparable tumor grade in SHAM, OVX and OVX+AI groups (Figure 3B). Analysis of TRAP stained sections also revealed no differences in osteoclast numbers per bone surface, further confirming a similar extent in tumor-induced bone erosion between SHAM and estrogen-depleted animals (Figure 3A and 3C). Thus, while ERα+/PR+ SSM2 and SSM3 cells retain hormone-dependent growth in the mammary fat pad [14], our findings demonstrate that SSM2 cells become resistant to ovarian hormone-deprivation once they are in the bone microenvironment.

### Established SSM3 bone tumors regress in the absence of estrogen

To determine whether established SSM3 tumors require estrogen to maintain their growth in the bone environment, we ovariectomized mice two weeks after SSM3 intra-tibial inoculation (herein defined as OVX post-IT), and determined the extent of bone erosion by micro- CT and tumor growth by histological analysis. To prevent any possible contribution of local estrogen synthesis, we also administered the aromatase inhibitor Letrozole to the OVX mice (OVX+AI post-IT). As positive control, SSM2 were also injected in the tibias of mice 2 weeks before OVX and aromatase inhibitor treatment.

**Figure 4:**
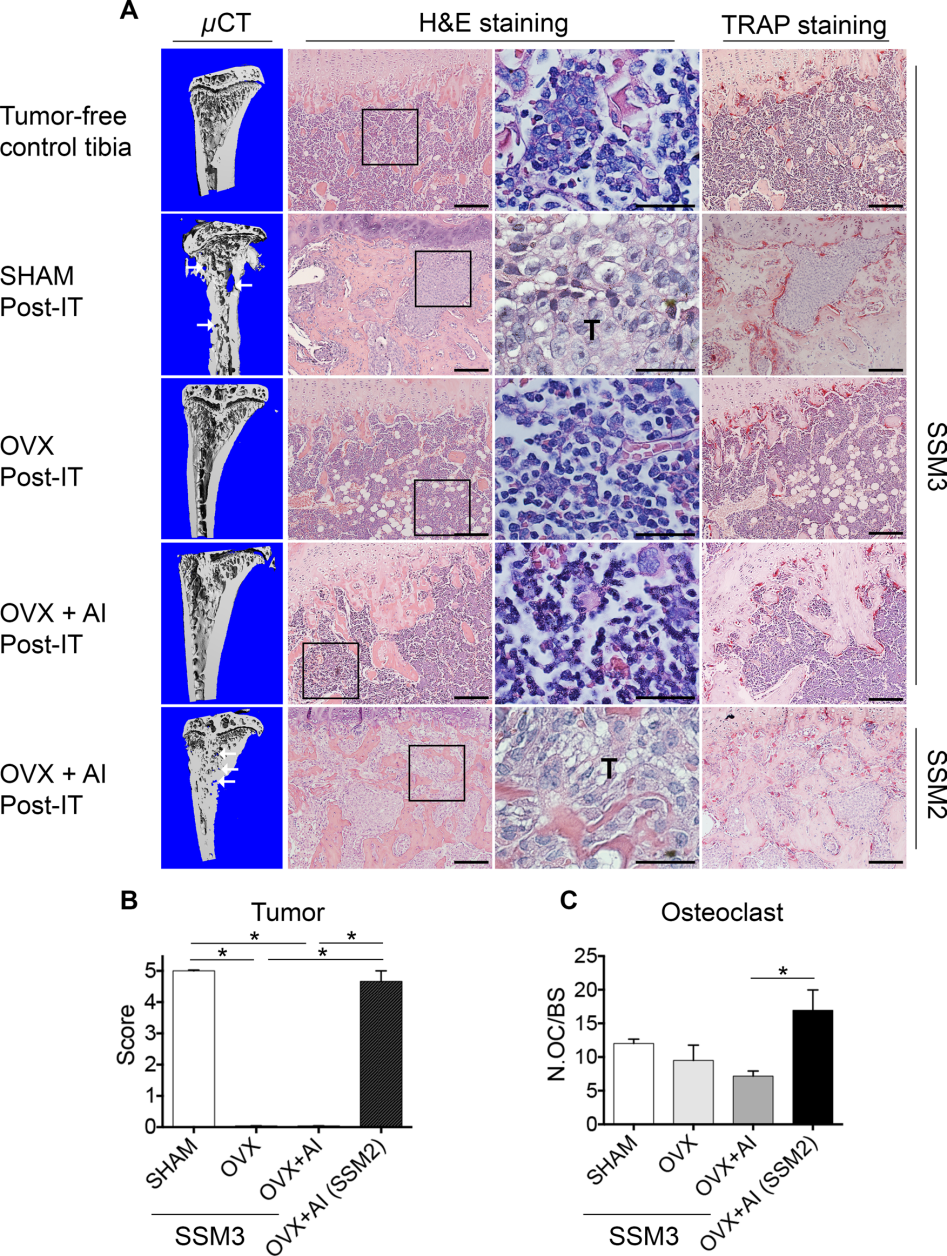
Established ERα+/PR+ SSM3 tumors are rejected following estrogen-deprivation (**A**) 10^5^ SSM3 cells were injected into the right tibia of WT female mice (*n* = 3/group), 2 weeks before SHAM- or OVX- surgery. One group of mice injected with SSM3 received the aromatase inhibitor Letrozole (AI) for 4 weeks post-OVX surgery. As control of tumor growth, mice injected with SSM2 cells were also subjected to OVX + AI. Representative images of micro-CT, H&E and TRAP staining sections (original magnification, 10× for H&E and Trap staining, scale bar = 100 μm. 60× magnification for H&E, scale bar = 25 μm). Arrows pointing to osteolytic lesions. Presence of SSM2 or SSM3 tumor cells is indicated by T. Representative H&E and TRAP staining is shown on sections from tumor-free tibia as control. (**B**) Tumor grade score from 0 to 5 showing displacement of marrow by tumor, with 0 indicating no tumor and 5 100% tumor in the bone marrow. Data are represented as mean +/− sem for SHAM, OVX, and OVX + AI SSM3 groups, as well as OVX + AI SSM2 group. *p < 0.05. (**C**) Number of osteoclasts (OC) per bone surface is shown for SHAM, OVX and OVX + AI SSM3 groups, and OVX + AI SSM2 group. Data are represented as mean +/− sem. *p < 0.05.

While osteolytic lesions were visible in tibias inoculated with SSM3 and subjected to SHAM post-IT surgery, we did not detect signs of bone erosion in all the other groups inoculated with SSM3 (Figure 4A). Histological sections confirmed that SSM3 grew only in the SHAM post-IT group, while evidence of tumor regression were visible in OVX post IT and OVX+AI post-IT (Figure 4A and 4B), indicating that SSM3 cells require estrogen to establish and maintain tumor growth in bone. By contrast, lytic lesions and extended tumor dissemination were observed in the SSM2 OVX+AI post-IT mice (Figure 4A, 4B). Surprisingly, the number of osteoclasts in the tumor bearing mice was similar between all SSM3-inoculated groups. Although SSM2 OVX + AI post-IT group showed higher osteoclast number compared to SSM3 OVX + AI post-IT group, no statistical differences were observed between the groups with tumor (ie SSM3 SHAM and SSM2 OVX + AI post-IT groups). These results suggest that SSM3 and SSM2 cell growth in bone does not rely on increased osteoclast recruitment or activity (Figure 4C).

In support of these findings, osteoclast precursors cultured *in vitro* in the presence of exogenous RANKL and M-CSF and exposed to conditioned media collected from SSM2 or SSM3 tumor lines gave raise to similar numbers of mature osteoclasts (not shown). No changes were also noted in the ability of the osteoblastic cell line ST2 to express alkaline phosphates following incubation with SSM2 or SSM3 tumor conditioned medium (not shown).

These data indicate that the different sensitivity to estrogen deprivation does not depend on changes to bone residing cells, suggesting existence of intrinsic differences between the two tumor lines.

### SSM2-hormone resistance is not due to loss of ER/PR expression

**Figure 5:**
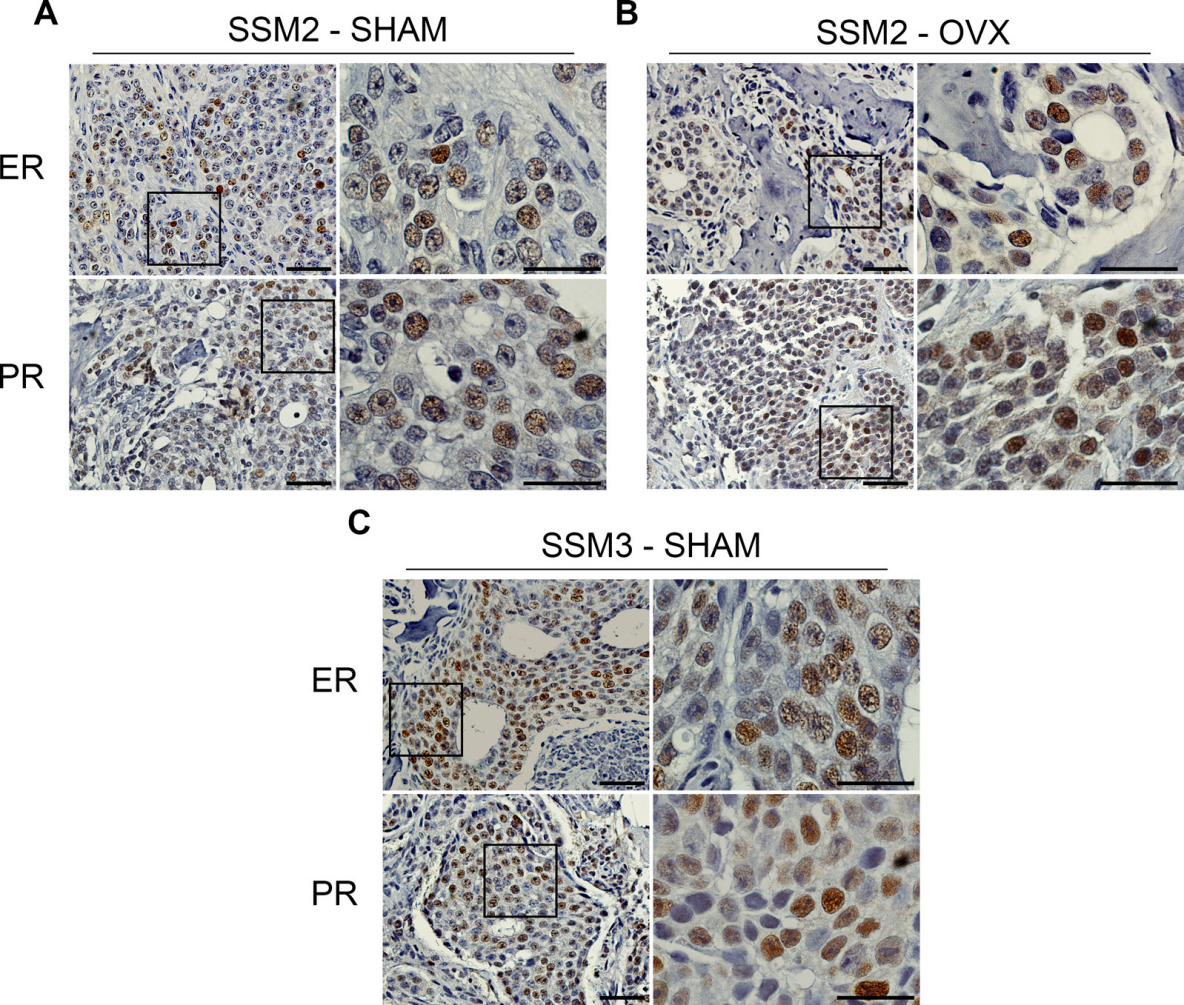
Bone residing SSM2 and SSM3 cells express ER and PR (**A–C**) SSM2 and SSM3 tumors in tibias of SHAM-operated (A, C) or OVX (B) female mice were subjected to immunohistochemistry to detect ER and PR expression. Representative images (original magnification, 20×. Scale bar = 50 μm. Insets, 60× magnification, scale bar = 25 μm) from 1 out of 5 mice per group are shown.

To understand how SSM2 cells survive to estrogen-deprivation in bone, we stained bone residing SSM2 and SSM3 cells for ERα and PR protein expression. We found that SSM2 tumor cells retained ERα and PR expression in both SHAM and OVX-operated mice (Figure 5A, 5B). Similarly, ERα and PR protein expression was observed in SSM3 bone tumors of SHAM-operated animals (Figure 5C). Therefore, downregulation of ERα and/or PR signaling or selection of ERα-negative SSM2 tumor cells is not the main mechanism used by SSM2 cells to survive in the absence of ovarian hormones in the bone microenvironment.

### Activation of JAK2/STAT3 is observed in both SSM2 and SSM3 cells growing in bone

**Figure 6:**
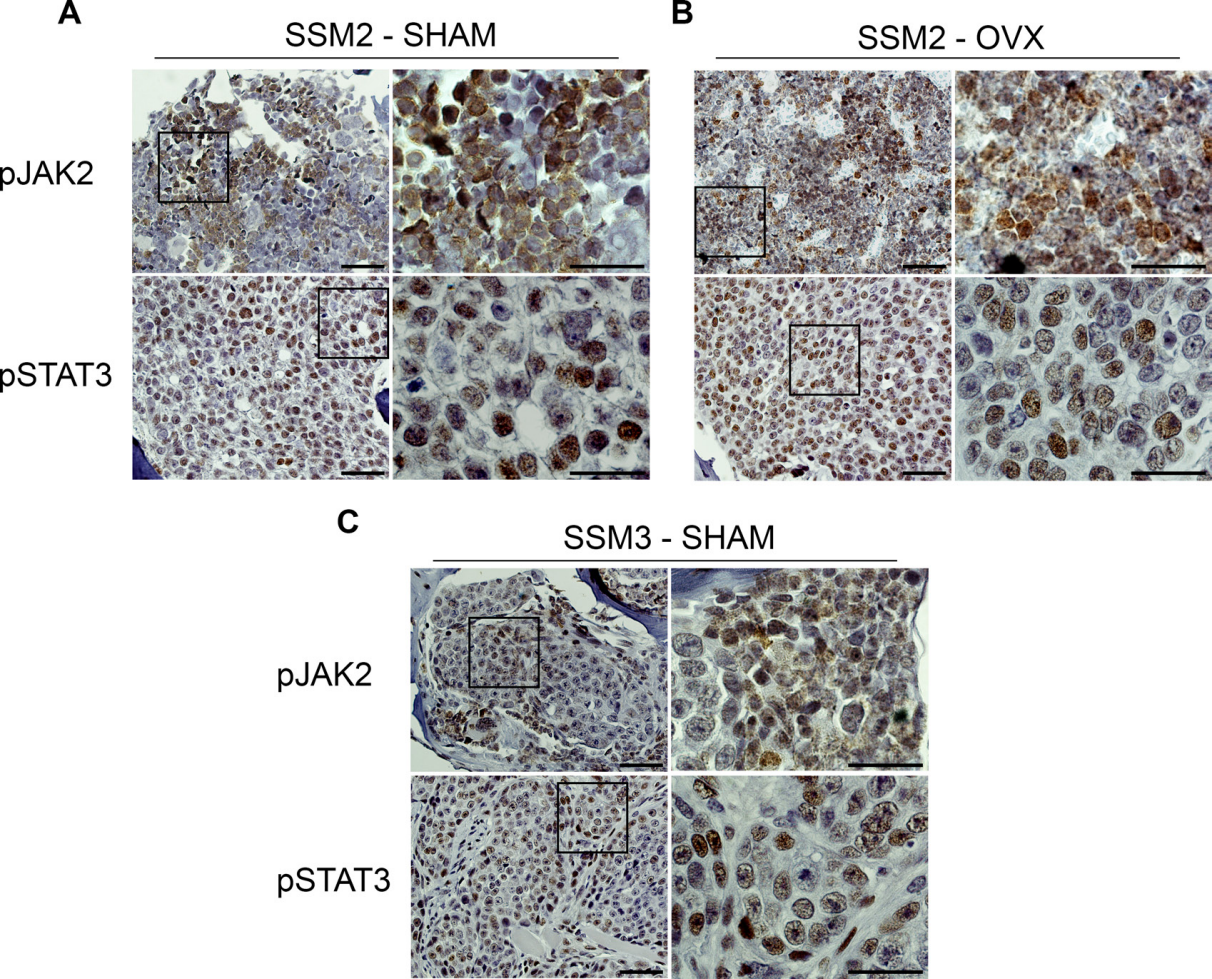
Bone residing SSM2 and SSM3 cells express pJAK2 and pSTAT3 (**A–C**) SSM2 and SSM3 tumors in tibias of SHAM-operated (A, C) or OVX (B) female mice were subjected to immunohistochemistry to detect pJAK2 and pSTAT3 expression. Representative images (original magnification, 20×. Scale bar = 50 μm. Insets, 60× magnification, scale bar = 25 μm) from 1 out of 5 mice per group are shown.

Activation of the prolactin receptor (PrlR) and JAK2-STAT3 signaling pathway persists in ERα+/PR+ breast tumors to provide a critical survival signal during tumor recurrence [16–19]. Furthermore, JAK2-STAT3 activation was shown to confer ovarian hormone independency in mice with established STAT1−/− breast tumors undergoing OVX [20]. To determine whether bone residing ERα+/PR+ SSM2 cells display activated PrlR signaling, we stained SSM2 tumors in bone from SHAM-operated or OVX mice for the phosphorylated forms of JAK2 (pJAK2) and STAT3 (pSTAT3). Similar pJAK2/pSTAT3 expression was observed in SSM2 bone tumors from SHAM and OVX mice (Figure 6A, 6B). Interestingly, pJAK2 and pSTAT3 levels were also detected in SSM3 bone tumors from SHAM-operated mice (Figure 6C). This result suggests that activation of PrlR signaling pathway is unlikely to be driving SSM2 hormone-independent growth in bone.

### ERα+/PR+ SSM2 mammary tumor cells do not develop metastases to lung in OVX mice

**Figure 7:**
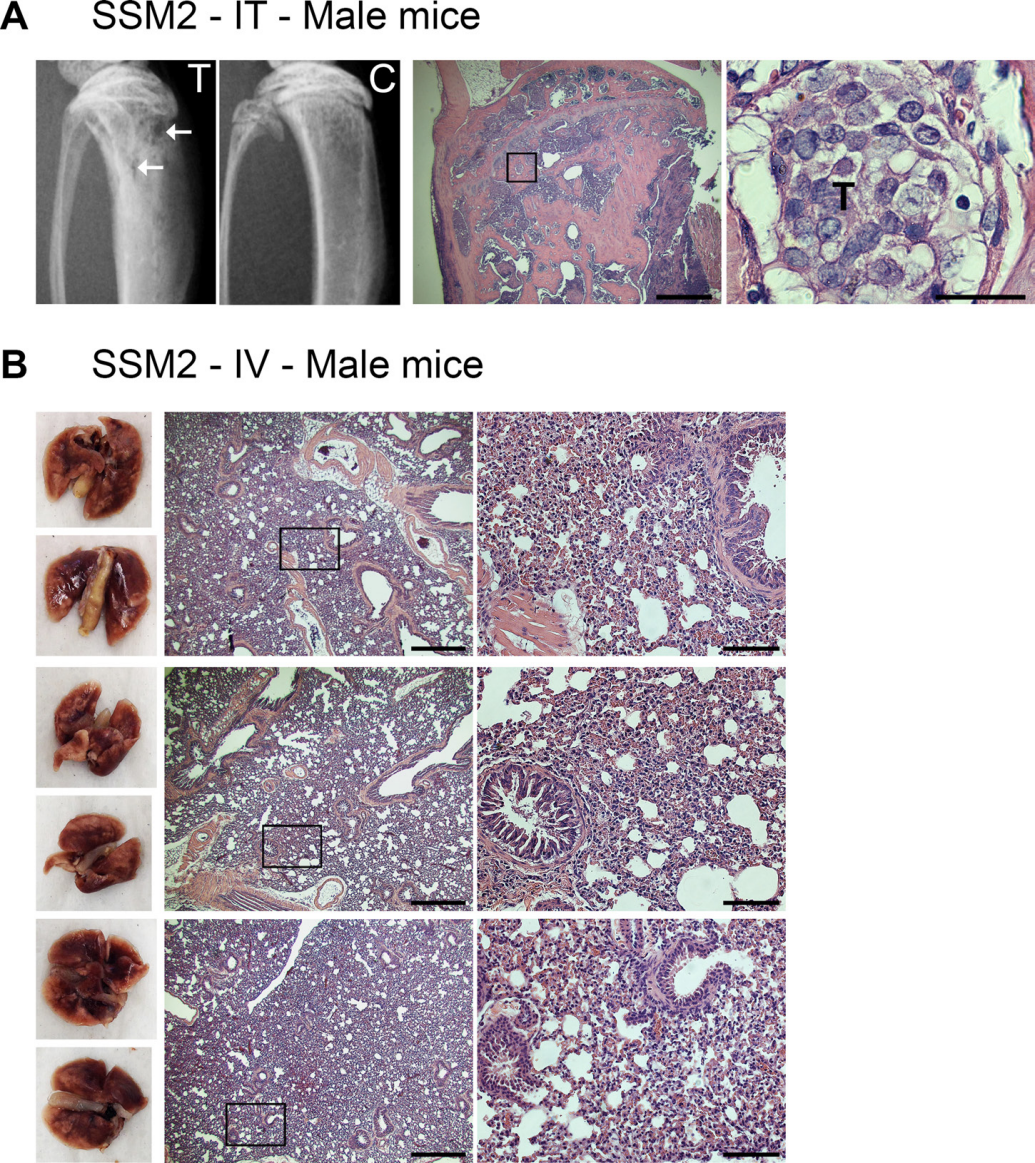
ERα+/PR+ SSM2 cells establish bone but not lung metastasis in male mice (**A**) 10^5^ SSM2 cells were injected into the right tibia (IT) of WT male mice (*n* = 5). Mice were sacrificed 30 days post tumor inoculation. Representative X-ray images of the tumor-injected tibia (T) showing lytic lesions (arrows) and non-injected control tibia (C) are shown. Presence of tumor cells in bone is depicted by T on histological sections stained for H&E (original magnification, 2×. Scale bar = 500 μm. Insets showing the presence of SSM2 tumor cells at 60× magnification, scale bar = 25 μm). (**B**) 5 × 10^5^ SSM2 cells were injected into the tail vein (IV) of WT male mice (*n* = 5). Mice were sacrificed 30 days post tumor inoculation. 3/5 representative images of fixed lungs and histological sections stained for H&E are shown (original magnification, 2×. Scale bar = 500 μm. Insets, 10× magnification, scale bar = 100 μm).

Besides the skeleton, lung is another predominant site of breast cancer metastasis [4]. Since ERα+/PR+ SSM2 tumor cells were capable of growing at skeletal sites in the absence of ovarian hormones, we next wondered if they could also develop lung metastasis in estrogen-deprived animals. As a consequence of reduced estrogen levels, ovariectomy enhances osteoclast activation and bone turnover, which is known to create a favorable environment for tumor colonization and proliferation in the bone microenvironment [21, 22]. Thus, to avoid any manipulation that would skew metastatic dissemination towards skeletal sites instead of lungs, we chose to use male mice, which supported SSM2, but not SSM3, growth in bone similarly to OVX animals (Figure 2E, 2F). 5 × 10^5^ SSM2 cells were injected in the tail vein of male animals to study tumor dissemination to lungs. As positive-tumor control, another group of male mice received 10^5^ SSM2 cells directly into the right tibias, to examine tumor growth in bone. After 30 days, SSM2 tumors were established in bone as determined by histology and presence of osteolytic lesions detected by X-rays (Figure 7A). By contrast, lung metastases were not detected in animals receiving intravenous SSM2 tumor cell inoculation (Figure 7B). Differently from the bone microenvironment, this result demonstrates that in the absence of ovarian hormones, the lung microenvironment is not capable of providing hormone-independent growth support of SSM2 cells.

### ERα+/PR+ SSM2 breast tumor cells do not support the growth of ERα+/PR+ SSM3 cells in the skeleton

**Figure 8:**
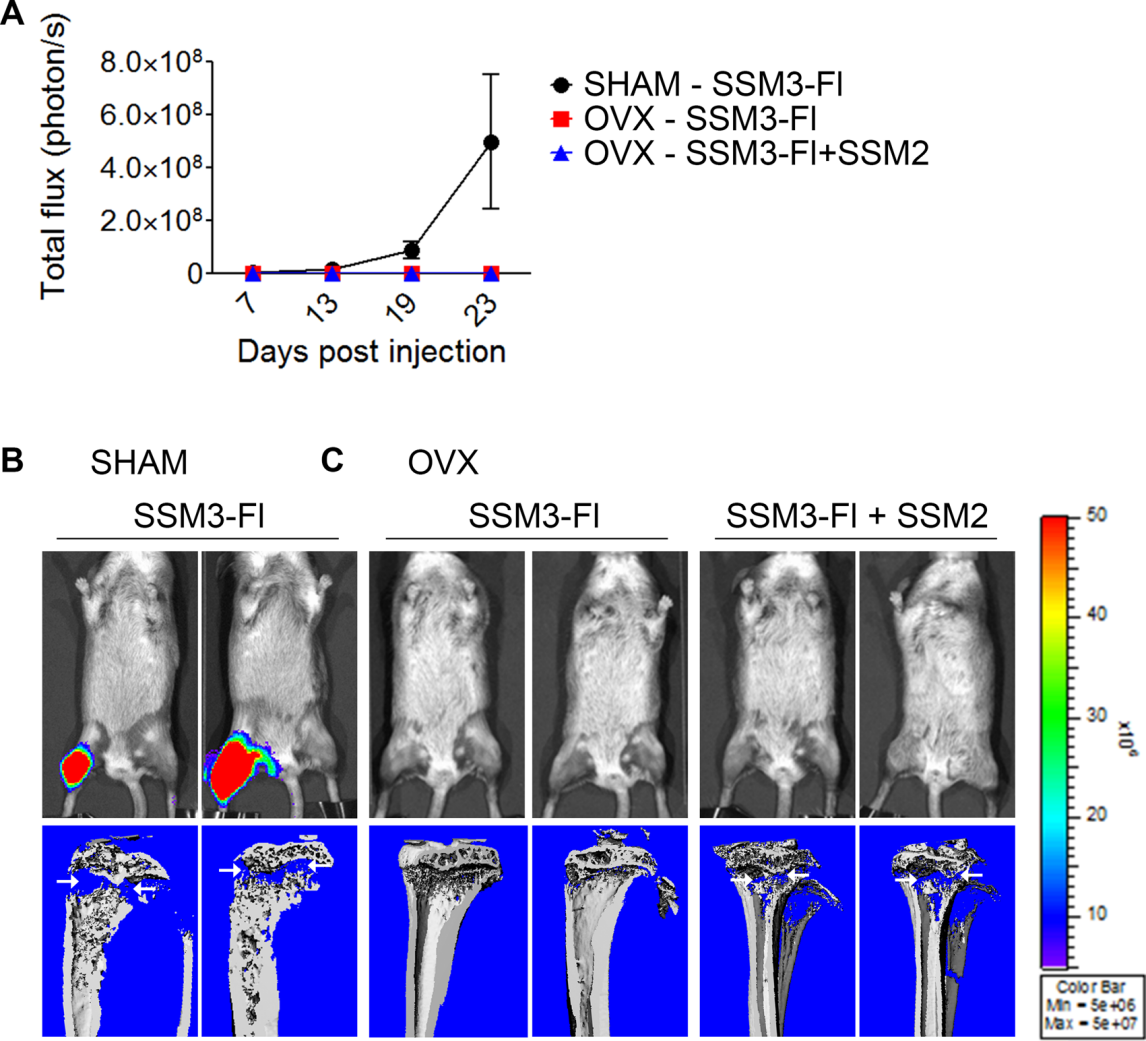
Bone preconditioning by SSM2 cells is not sufficient to allow estrogen-independent growth of SSM3 cells (**A–C**) 10^5^ SSM2 and 10^5^ SSM3-Fl cells were co-injected into the right tibia of WT female mice, one week after OVX surgery (*n* = 6). SHAM-operated and OVX mice were injected with 10^5^ SSM3-Fl cells into the right tibia alone and used as controls (*n* = 6/group). (A) Bioluminescence imaging monitoring SSM3-Fl tumor growth (mean +/− sem) in SHAM-operated mice (black circles) and OVX mice (red squares for SSM3-Fl alone and blue triangles for SSM3-Fl + SSM2 cell co-injection). (B, C) 2 out of 6 representative bioluminescence images and viva-CT scans of the right tibias of SHAM-operated (B) and OVX (C) mice from (A) at 27 days post tumor injection are shown. Osteolytic lesions are depicted by arrows.

To determine whether SSM2 cells can precondition the bone microenvironment to allow ovarian hormone-independent growth of SSM3 cells, we co-injected 10^5^ SSM3 cells conjugated with firefly luciferase (Fl) and 10^5^ unlabeled SSM2 cells into the right tibias of OVX mice. As positive and negative control, 10^5^ SSM3-Fl cells alone were injected into the tibias of SHAM-operated or OVX mice, respectively. Detection of bioluminescence signal would indicate growth of SSM3 cells. As expected, SSM3- Fl cell grew in SHAM-operated animals and induced osteolytic lesions (Figure 8A, 8B). However, we were unable to detect any bioluminescence signal in OVX mice injected with SSM3-Fl alone or together with SSM2 cells (Figure 8A). Nevertheless, lytic lesions were detected in bones co-injected with both tumor cell lines (Figure 8C), confirming estrogen-independent growth of SSM2 cells. This result suggests that ERα+/PR+ SSM2 breast tumor cells harbor intrinsic genetic differences that allow them to grow in the skeleton in an estrogen-independent manner.

## DISCUSSION

To our knowledge, herein we describe the first clinically relevant ERα+/PR+ murine breast cancer model of hormone independence in the context of bone metastases in immunocompetent mice. In this study we have used SSM2 and SSM3 ERα+/PR+ murine cell lines derived from spontaneous breast carcinomas in mice lacking STAT1, a transcription factor associated with tumor progression in ERα+ breast cancer patients [14]. We now show that, in the bone microenvironment, SSM2 cells grow independent of ovarian hormones, while SSM3 growth remains estrogen-sensitive. Intriguingly, SSM2 cells do not engraft at primary site and in the lungs in the absence of estrogen. Thus, SSM2 cells represent a clinically relevant tumor model of estrogen-independent growth at skeletal sites.

Several studies have used ERα+/PR+ human breast cancer cell lines, which can only grow in immunocompromised mice, to study mechanisms of resistance to anti-hormone therapies. These models provide important insights into genetic changes within tumor cells that confer estrogen resistance. However the use of these tumor lines limits our understanding of the possible effects of the immune system in the acquisition of tumor estrogen-independency. Studies aimed at understanding the mechanisms of breast cancer bone metastases have largely utilized the highly metastatic ERα- MDA-MB-231 human breast cancer cell line. While offering a great tool to study mechanisms of tumor growth in bone, this model cannot provide any insights into the hormone dependence/independence of ERα+ tumors. Considering that patients with ERα+ breast tumors often develop recurrences at skeletal locations, the current tumor models cannot replicate this clinical scenario.

It has been proposed that ERα+ cancer cells may stay dormant in the bone microenvironment until specific signals awaken them, thus contributing to metastatic relapse. Several mechanisms have been proposed to explain how the cancer cells survive in the bone marrow, including immunosurveillance, angiogenic switch and interaction with extracellular matrix and stromal cells [23]. We and others have shown that accumulation of myeloid populations with immune suppressive effects increases the rate of bone metastases [24–26]. Therefore, a useful tumor model of ERα+ breast cancer resistant to anti-hormone therapies at skeletal sites, should consider the interactions between the bone microenvironment, the immune system and ERα+ mammary tumor cells. Although mechanistic insights gained from studying the murine mammary SSM2 tumor cells will have to be validated in human tumors, SSM2 cells represent an important tumor model that may help define new therapeutic targets for breast cancer patients with skeletal recurrences.

The majority of women who relapse on aromatase inhibitors continue to express functional ERα, which remains a legitimate target for second-line endocrine therapy [10]. There are several lines of evidence that tumors retaining ERα expression may escape the restraints of estrogen-deprivation by increasing their sensitivity to residual estrogens and re-activating ERα signaling. *In vitro*, MCF-7 cells adapted to long-term estrogen-deprivation require less estrogen for maximal growth stimulation compared to parental cells [27]. This adaptation, driven by the selection pressure of estrogen-deprivation, involves increased ERα expression and alterations in cross-talk with growth factor signaling pathways or various cytokines. Activation of MAPK/ERK, AKT, or p38MAPK can sensitize ERα to activation by estrogen (resulting in hypersensitivity) [27], and/or activate ERα in the absence of estrogen (resulting in estrogen-independence). In our model, we show no changes in ER/PR expression in SSM2 bone tumors in SHAM versus OVX animals. Furthermore, the observation that SSM2 cells are dependent on estrogen at the orthotopic location or at other metastatic sites such as the lung but not in bone, suggests that ERα+/PR+ SSM2 cells are likely to receive specific survival and proliferative signals from the bone microenvironment. This result is important as it strengthens the hypothesis that the interplay between tumor cells and the host environment, e.g. bone cells, drives resistance to hormone deprivation and tumor relapse. However, in contrast to SSM2, ERα+/PR+ SSM3 cells require ovarian hormones to survive and proliferate in the bone, similarly to the mammary fat pad location [14], suggesting intrinsic genetic differences between SSM2 and SSM3 cell lines. This assumption is further supported by our observation that preconditioning the bone microenvironment by injecting SSM2 cells together with SSM3 cells fails to support estrogen-independent growth of SSM3 in OVX mice. Thus, our tumor models recapitulate the heterogeneity of breast cancer patients with ERα+ tumors, some of which acquire resistance to anti-hormonal therapies while others are sensitive to the same treatments.

Nevertheless additional questions remain. What are the signaling pathways that drive SSM2 cell proliferation independent of estrogen in bone? We have recently shown that both ERα+/PR+ SSM2 and SSM3 mammary fat pad tumors regress following ovariectomy [20]. However, recurrence can occur at the same mammary location when ovariectomy is performed after the tumors are established, suggesting that the two tumor cell lines are capable of adapting to estrogen-deprivation at the primary site [20]. Our previous finding demonstrated that SSM2 and SSM3 cells display persistent prolactin receptor signaling with activation of JAK2, STAT3 and STAT5A/5B, and that pharmacological inhibition of pJAK2 increased SSM2 and SSM3 tumor cell apoptosis [20]. This result suggests that prolactin receptor signaling through JAK-STAT activation provides a survival signal to ERα+/PR+ SSM2 and SSM3 cells and allows them to grow in the absence of estrogen. Similar to the orthotopic location, we now find that SSM2 tumor cells growing in bones of OVX mice show phosphorylation of JAK2 and STAT3, suggesting activation of the PrlR signaling pathway in the absence of estrogen. However, both SSM2 and SSM3 tumor cells also express the activated forms of JAK2 and STAT3 in SHAM-operated mice. This result is in agreement with our previous study showing that inhibition of JAK2 activation as a first-line treatment causes primary tumor regression in OVX mice [20]. Therefore, although the prolactin pathway is active in both SSM2 and SSM3 bone tumors, PrlR signaling is not sufficient to mediate the growth of ERα+/PR+ SSM3 cells in bones in the absence of ovarian hormones. Furthermore, this result suggests the existence of different mechanisms of estrogen-resistance at the primary site and in bone.

Do SSM2 cells alter the bone microenvironment to support estrogen-insensitive growth or is it the bone microenvironment that changes the tumor cells thus promoting estrogen-independent tumor growth? Cells within bone, and osteoclasts in particular, play an important role in facilitating tumor cell colonization and expansion in the bone microenvironment. By releasing bone-stored factors, osteoclasts provide proliferating and survival signals for the cancer cells. In turn, bone residing cancer cells stimulate osteoclast activation and bone erosion thereby establishing a tumor/bone vicious cycle. Both SSM2 and SSM3 bone tumors induce osteolytic lesions, thus osteoclasts are activated by the presence of either SSM2 or SSM3 cells. In support of this hypothesis, we observed similar osteoclast numbers *in vivo* in mice intratibially injected with either SSM2 or SSM3 cells or *in vitro* when bone marrow cells were cultured in the presence of SSM2 or SSM3 conditioned medium. In addition, the co-injection of SSM2 and SSM3 tumor cells in bones of OVX mice did not allow estrogen-independent growth of SSM3 cells. Thus, osteoclast activation is not sufficient to induce hormone independent tumor growth in bone. Similarly, in the clinic ER+ breast cancer patients with tumor relapses are treated with anti-resorptive agents. However, while protecting from skeletal related events, osteoclast blockade does not seem to confer any increase in disease-free survival [7]. Our previous finding demonstrating that deficiency in anti-tumor T cell responses can overcome the anti-tumor effects of Zoledronic Acid in bone [26] suggests that interactions between tumor cells and the immune system may also contribute to the ability of SSM2 cells to grow in bone in the absence of ovarian hormones. At the moment, the relative contribution of genetic changes within the tumor cells and the microenvironment to hormone-independent growth of SSM2 is not clear. Future inquiries aimed at examining which pathways are uniquely activated in SSM2 tumor cells and how bone residing cells contribute to facilitating estrogen-independent growth of SSM2 cells would help the design of better therapies aimed at targeting ER+ tumors refractory to anti-hormone therapies.

In conclusion, we developed a unique ERα+/PR+ murine breast cancer model that is highly relevant to the clinic and will help understanding mechanisms of hormonal resistance in the context of skeletal metastases.

## MATERIALS AND METHODS

### Mice

Wild-type 129S6/SvEv (WT) mice were purchased from Taconic Farms (Hudson, NY, USA). Animals were housed in a pathogen-free animal facility at Washington University. 6-8-wk-old littermate mice were used in all experiments according to protocols approved by the Institutional Animal Care and Use Committee.

### Cell cultures

STAT1−/− mammary tumor cell lines, SSM2 and SSM3, were originated from two individual STAT1−/− tumor-bearing mice [14]. Briefly, spontaneous tumors were mechanically disaggregated before an overnight incubation with collagenase. Stromal fibroblasts were eliminated by differential trypsinization. The absence of fibroblasts was determined by immunofluorescence using an antibody specific for vimentin [14]. The SSM cell lines were cultured at 37°C with 5% CO2 in DMEM/F12 supplemented with 10% FBS, 1% L-glutamine, 1% penicillin-streptomycin, 50 μM 2-mercaptoethanol, 0.3 μM hydrocortisone, 5 μg/ml insulin and 10 ng/ml transferrin.

### Tumor models

To establish bone tumors, 10^5^ SSM2 or SSM3 cells were directly injected into the tibia of WT male or female mice. When indicated, female mice were either SHAM-operated or ovariectomized (OVX) under general anesthesia. 10^5^ SSM2 or SSM3 cells were intratibially injected either one week post-surgery or two weeks pre-surgery. Animals were fed an estrogen-free diet throughout the duration of the experiments (Harlan Teklad, Madison, WI, USA). In some experiments, animals received the aromatase inhibitor Letrozole (TOCRIS Bioscience) at 10 ug/day in addition to OVX surgery, for 4 weeks.

To establish lung metastases, 5 × 10^5^ SSM2 cells were injected into the tail vain of WT male mice. Another group of WT male mice received 10^5^ SSM2 cells into the right tibias and were used as control for tumor growth. Animals in both groups were euthanized 30 days post tumor inoculation.

### Bioluminescence imaging

SSM3 cells were virally transduced with FUGW plasmid to express firefly luciferase. The resulting cells are referred to as SSM3-Fl. 10^5^ SSM3-Fl cells were injected alone or in combination with 10^5^ non-labelled SSM2 cells. Tumor growth in bone was monitored on days 7, 13, 19 and 23 by bioluminescence imaging (BLI) using an IVIS 100 imaging system (Caliper Life Sciences). Bioluminescence photon flux (photons per second) data were analyzed by region of interest measurements in Living Image 3.2 (Caliper Life Sciences).

### Radiography, computed-tomography (CT) and histology

Radiographic analyses were performed on a Faxitron X-ray machine every other week and bone erosion was confirmed by viva-CT (Scanco Medical). 3D images from intact mouse tibia were obtained on a micro-CT40 scanner (Scanco Medical).

Four-micron sections of fixed, decalcified, paraffin-embedded long bones were histochemically stained with hematoxylin and eosin (H&E) to observe tumor cells. When indicated, two blind operators graded bone sections from 0 to 5 based on the extent of displacement of normal marrow by tumor, with 0 indicating no tumor, 1 0–25%, 2 25–50%, 3 50–75%, 4 75–100% and 5 100% tumor occupying the marrow space. To examine the presence of ERα and PR, slides were deparaffinized, serially rehydrated, and stained with antibodies against ERα (mouse monoclonal antibody 6F11; 1:50, Leica, Wetzlar, Germany), PR (rabbit polyclonal antibody; 1:100, Dako, Carpinteria, CA, USA), the phosphorylated form of JAK2 (pJAK2, rabbit monoclonal E132; 1:200, Abcam, Cambridge, MA, USA), and the phosphorylated form of STAT3 (pSTAT3, rabbit monoclonal D3A7; 1:80, Cell Signaling, Boston, MA, USA).

### Statistical analysis

Statistical analysis was performed using one-way ANOVA with a Tukey post -test, with Prism software (GraphPad). The *p* values < 0.05 were considered as significant.
